# The Sanitary Blocks of Hospitals

**Published:** 1920-03-27

**Authors:** 


### HOSPITAL CONSTRUCTION: THE NEW ERA.

### The Sanitary Blocks of Hospitals.

We have in a previous paper touched on the subject of the sanitary blocks of hospitals. A special interest has recently been aroused in this topic by a signed article contributed to a contemporary by Mr. Keith D. Young. Mr. Young's experience in hospital design is so extensive and so long that anything he may say on the details of planning carries weight and influence. It is on these grounds that we find ourselves minded to say a few words of criticism upon the arguments he brings forward. Mr. Young anticipates the criticism, for he admits that his proposals are bold, and expects that they will be met by adverse comment.

Briefly Mr. Young's argument is as follows. In the dark ages of sanitation that truly bright light, Florence Nightingale, laid it down as a principle that the sanitary conveniences necessary to a ward should in all cases be built externally to the building, and cut off by a lobby separately-lighted and ventilated by cross-windows. Mr. Young points out that the w.c. of Florence Nightingale's day was so abominable a thing that its exclusion from the atmosphere of the ward was obviously essential. But he claims that the modern apparatus, attached as it is to modern plumbing and modern drainage, is worthy of a more cordial welcome and of greater confidence. There is no doubt that the views of sanitarians and of modern medical men have undergone a great change in the past twenty years. Time was when a wash-hand basin was under suspicion in a ward. Now, of course, neither wards nor operating theatres are without them. As to baths we are beginning to admit them within the atmospheric cordon of the sick-room, and our architects are realising with relief the abolition of the law which used to confine the bath to the "cut-off" region. But even the bath should not abuse our hospitality. For some reason or other hospital after hospital has been built in these enlightened after-years in which no provision has been made for washing bed macintoshes. The result is that there prevails a general practice of washing these appliances in the patients' baths. It is a nasty expedient, and should be provided against by the provision of proper large sinks for the purpose, and of fixed rails on which the macintoshes can be dried.

But supposing that all is properly designed, and we admit the bath to the ward *enceinte*, there remain the sink room (sluice room or bed-pan room) and the water-closets to be provided for. Shall these things, for the sake of obvious economy and equally obvious convenience, be permitted to be placed in a passage just outside the ward, separated from the ward only by a door, and unprovided with the traditional barrier of two doors with two cross-draught windows between them?

Mr. Young says "yes"; and he says it with admirable courage. He says or implies that we have got to trust our plumbing, and he also says that, even if we provide the two doors and the two windows, nurses will contrive either to set the two former open, or to keep the two latter shut, or both.

Perhaps a story of practical experience will throw some light on this problem. An architect recently visited a small and attractive hospital in which some alterations were contemplated. Entering the women's ward first he found adjoining it a suite of sanitary rooms arranged exactly on the principle that Mr. Young recommends. They opened into a corridor leading direct from the ward, which corridor was ventilated by a skylight. Everything about this sanitary group was clean, well made and up-to-date. Money had been freely spent on tiles, enamel paint, impervious floors, and first-rate fittings. In fact the impression produced led at once to the conclusion that it would be mere pedantic dogmatism to condemn the arrangements on the sole ground that our old friend the "cut off" was missing. The next visit paid was to the men's ward. This lay on the west flank of the hospital, and a westerly wind was blowing. A touch on the swing-doors leading to the sanitary block resulted in their being blown forcibly inwards towards the ward (and even when they were closed again it was found that the draught under the doors was a very strong one). Examination of the sanitary rooms showed, as on the women's side, that the utmost care had been taken to provide perfect appliances. Examination also showed that the strong current of air was coming from the window of the sink room.

The sink room, like the rest of the arrangements, was quite modern and well appointed. On enquiry it was found that an indraught at the corner of the ward nearest the door to the sanitary group was habitual, and that patients did not care for that part of the ward.

In fact it was established that on the particular date of the visit virtually the whole air-supply of the ward was passed in over a bed-pan sink, and over that little collection of specimens awaiting inspection which one would not generally select as the purifying agency for an air inlet.

Now is this a risk worth running? We think not. The only way of counteracting a strong and constant wind pressure from an undesirable quarter is to give the wind a ready and innocuous egress. Such an egress is easily obtained by a "cut-off" window on the leeward side. No shutting of the bed-pan room door would have made the room draught-proof, no mere skylight ventilator in the passage would have counteracted the wind's temptation to join forces with the fire-suction in the ward. But

Previous articles appeared on Jan. 25, Feb. 8, March 1, 15, 29, April 19, May 17, June 14, July 19 Aug. 23, Sept. 20 and Oct. 25, 1919, and Jan. 10, p. 333.

**The New Era**—(*continued*).

an inlet and outlet in a cut-off passage, protected by a door each side, would undoubtedly have diverted the cheery breeze which was so innocently making its way from the open air of heaven to the warmth of the ward through a space which was at least open to suspicion.

In America, where most of the ventilation is artificial, and in those unhappy institutions of our own country, where a *plenum* system prevails, it may be all very well to trust to the hope that the air current will habitually be *from* the ward and *toward* the sanitary group, but when all is said and done it remains a fact that in our country, where we generally rely on natural air currents, it is foolish to run the risk of liberally feeding our wards with air passed through these sanitary groups.

Mr. Young suggests that the corridor can be kept ventilated and cross-ventilated by making the ceilings of the w.c.s, etc., lower than that of the corridor, and placing windows above the roof of the former. This is true, but it is only a half-way expedient.

We are whole-heartedly in favour of testing everything in the light of the most modern knowledge, but we cannot yet make up our minds to endorsing any building device which involves the risk of submitting patients to the opportunity of habitually breathing the air of the region set apart for human refuse.

